# Dietary advanced glycation end products are associated with an increased risk of non-alcoholic fatty liver disease in Iranian adults

**DOI:** 10.1186/s12902-023-01365-8

**Published:** 2023-05-18

**Authors:** Mitra Kazemi Jahromi, Asal Neshatbini Tehrani, Farshad Teymoori, Ghazal Daftari, Hamid Ahmadirad, Niloufar Saber, Ammar Salehi-Sahlabadi, Hossein Farhadnejad, Parvin Mirmiran

**Affiliations:** 1 Endocrinology and Metabolism Research Center, Hormozgan University of Medical Sciences, Bandar Abbas, Iran; 2 Student Research Committee, Ahvaz Jundishapur University of Medical Sciences, Ahvaz, Iran; 3 Department of Nutrition, School of Allied Medical Sciences, Ahvaz Jundishapur University of Medical Sciences, Ahvaz, Iran; 4 Nutrition and Endocrine Research Center, Research Institute for Endocrine Sciences, Shahid Beheshti University of Medical Sciences, Tehran, Iran; 5 Department of Nutrition, School of Public Health, Iran University of Medical Sciences, Tehran, Iran; 6 School of Medicine, Tehran University of Medical Sciences, Tehran, Iran; 7 Department of Clinical Nutrition and Dietetics, Faculty of Nutrition and Food Technology, National Nutrition and Food Technology Research Institute, Shahid Beheshti University of Medical Sciences, Tehran, Iran; 8 Department of Community Nutrition, School of Nutrition and Food Science, Isfahan University of Medical Sciences, Isfahan, Iran

**Keywords:** Dietary advanced glycation end products, Dietary pattern, Liver disease, Non-alcoholic fatty liver diseases, Adults

## Abstract

**Background:**

Dietary advanced glycation end products(AGEs) may contribute to increased inflammation and oxidative stress as risk factors for chronic diseases such as liver disease. In the current study, we aimed to examine the possible association of dietary AGEs with the odds of non-alcoholic fatty liver disease (NAFLD) in Iranian adults.

**Methods:**

A total of 675 participants (225 newly diagnosed NAFLD cases and 450 controls), aged  20–60 years, were recruited for this case-control study. Nutritional data were measured using a validated food frequency questionnaire, and dietary AGEs were determined for all participants. An ultrasound scan of the liver performed the detection of NAFLD in participants of the case group without alcohol consumption and other causes of hepatic disorders. We used logistic regression models, adjusted for potential confounders, to estimate the odds ratios(ORs) and 95% confidence interval(CI) of NAFLD across tertiles of dietary AGEs.

**Results:**

Mean ± SD age and body mass index of the participants were 38.13 ± 8.85 years and 26.85 ± 4.31 kg/m^2^, respectively. The median(IQR) of dietary AGEs in participants was 3262(2472–4301). In the sex and age-adjusted model, the odds of NAFLD were increased across tertiles of dietary AGEs intake(OR:16.48;95%CI:9.57–28.40, P_trend_<0.001). Also, in the final model, after controlling for confounding effects of BMI, smoking, physical activity, marital status, socio-economic status, and energy intake, the odds of NAFLD were increased across tertiles of dietary AGEs intake(OR:12.16; 95%CI:6.06–24.39, P_trend_<0.001).

**Conclusion:**

Our results showed that greater adherence to dietary pattern with high dietary AGEs intake was significantly related to increased odds of NAFLD.

## Background

Nonalcoholic fatty liver disease (NAFLD) is the major cause of liver disease worldwide [1]. This liver abnormality is related to the excessive accumulation of lipids in the hepatocytes in the absence of alcohol consumption or viruses [2, 3], which may progress to nonalcoholic steatohepatitis, fibrosis leading to cirrhosis, and hepatocellular carcinoma [4]. Genetic and epigenetic factors, the microbiome, dietary patterns, and adipokines affect NAFLD progression [5]. NAFLD prevalence depends on age distribution, geographic area, and risk factors varying from 33.6% in the USA [6], 25% in Asia [7], 25% in Europe [8], and a total of 15–30% in the general population [9]. A recent meta-analysis reported the prevalence of NAFLD in Iranian adults to be nearly 35%, which indicates the growing prevalence of this metabolic disorder in the Iranian population [10]. NAFLD is presumed to be linked to other metabolic abnormalities, including high blood glucose levels, central adiposity, insulin resistance, hypertension, and dyslipidemia [11].

A sedentary lifestyle and unhealthy diet [12], along with chronic disorders such as diabetes [13], obesity, cardiovascular disease [14], and hypertension [15], are the most causes of NAFLD. An unhealthy diet has been considered in relation to the increased risk of liver disease from various aspects in previous investigations. Dietary patterns rich in processed or red meat [16], high intake of sodium [17], and galactose are associated with NAFLD development [18]. Dietary advanced glycation end products (AGEs) are one of the other harmful food compounds in the food pattern, which can be important in the pathogenesis of diseases [19]. These dietary products are a heterogenous group produced nonenzymatically by the reaction between free amino groups of lipids, proteins, or nucleic acids with reducing sugar such as glucose [20]. The mechanisms of production include lipid peroxidation, the Maillard reaction, and the polyol pathway [21]. N-carboxymethyl lysine is the AGEs marker in foods rich in meat, meat-substitute, and fat groups [22]. Furthermore, cooking time, moisture, temperature, and the means of cooking affect the amount of AGEs in the foods [22].

Previous reports are reported that AGEs can contribute to increasing the level of inflammation and oxidative stress as risk factors related to chronic diseases, such as diabetes, atherosclerosis, cancers, obesity, insulin resistance, metabolic syndrome (MetS), and hypertension [19, 21]. A prospective study on Iranian adults suggested that higher adherence to a diet with limited dietary AGEs may prevent hypertension and its complications [23]. Also, a cross-sectional study of Mexican adults showed that a high intake of AGEs was significantly related to impaired fasting glucose and increased risk of MetS [24]. Furthermore, a review article revealed that AGEs are harmful to cardiovascular disease due to the oxidation of low-density lipoproteins, resulting in obesity and insulin resistance by increasing body weight and energy intake [25].

Although AGEs accumulation in animal models with hepatic steatosis initiates cellular dysfunction and apoptosis leading to NAFLD [26, 27], so far, in epidemiologic studies, the role of this harmful food compound in predicting the risk of NAFLD has not been investigated. Therefore, the current study is the first study that aimed to assess the possible association of the risk of AGEs intake with the odds of NAFLD in a sample of Iranian adults.

## Materials and methods

### Study population

The current hospital-based case-control study was carried out in the Metabolic Liver Disease Research Center a referral center affiliated to Isfahan University of Medical Sciences, and we used convenience sampling to recruit the participants (age range from 20 to 60 years). Six hundred seventy-five individuals were enrolled in the present study including 450 control and 225 new cases of NAFLD that their diagnosis was confirmed by an ultrasonography scan of the liver consistent with NAFLD without consumption of alcohol and other causes of liver disease. Also, control subjects were selected from healthy individuals with no stage of liver steatosis based on liver ultrasonography. Inclusion criteria include the following: (1) lack of specific diet that influences the patient’s weight; (2) lack of history of liver diseases, kidney diseases, cardiovascular disease, diabetes, thyroid disorder, malignancy, and autoimmune diseases, and do not taking take the potentially steatogenic or hepatotoxic medicines; (3) being in the range of 20–60 years. Also, the participants with under-reported or over-reported dietary intake (≤ 800 or ≥ 4500 kcal/day) and those who completed less than 35 items of the food frequency questionnaire (FFQ) were excluded (8 participants) and were replaced by other eligible subjects. The sample size for the current study was calculated using the following formula:

$$n = \left( {\frac{{1 + \varphi }}{\varphi }} \right)\frac{{({z_{1 - \frac{\alpha }{2}}} + {z_{1 - \beta }})}}{{{{(\log {\text{ }}OR)}^2}\overline \pi \left( {1 - \overline \pi } \right)}} = 225$$

z = 1.96, OR = 1.5 and prevalence (p) = 0.35. Controls were twice the case group.

### Dietary assessment

Subjects’ dietary intakes for cases (1 year before diagnosis) and controls (1 year before the interview) groups using valid and reliable 168-item semi-quantitative FFQ were collected by trained dietitians [28]. This questionnaire consisted of 168 food items of common Iranian foods with standard serving sizes. A trained dietitian asked participants to express their frequency of consumption of each food item over the last year by choosing one of the following classifications: “never or less than once a month”, “3–4 times per month”, “once a week”, “2–4 times per week”, “5–6 times per week”, “once daily”, “2–3 times per day”, “4–5 times per day”, and “6 or more times a day”. Portion sizes of all food items using standard Iranian household scales were converted to grams [29]. Also, the daily intake of energy, micronutrients, and macronutrients for each participant was calculated by the United States Department of Agriculture’s (USDA) Food Composition Table (FCT) [30]. For some traditional foods that Iranians consume and do not exist in USDA FCT, we used the Iranian FCT [31]. Then we transformed consumed food frequency into a daily intake scale.

The dietary AGEs scores were calculated using AGEs content in 108 food items of 168-FFQ based on the studies conducted by Goldberg et al. [22] and Uribarry et al. [32]. To measure this score, we calculated the AGEs content (kU/100 g) of each food item in our FFQ based on the above-mentioned studies. For some traditional foods, we considered similar items found in these development studies. Finally, we summed the AGEs value of all food items for every person as its AGEs score [23].

### Assessment of other variables

In addition to information on dietary intake, we measured anthropometric characteristics such as body height, body weight, and body mass index (BMI). A trained dietician measured the participant’s weight using a standard digital Seca scale (made in Germany) while participants wore minimum clothes and without shoes and recorded to the nearest 100 g. Height also was measured using a mounted tape meter in a standing relaxed shoulder position with no shoes to the nearest 0.5 cm. BMI was calculated as weight (kg) divided by height (*m*^2^). Information on age, sex, smoking status, marital status, and education level was collected using a standard questionnaire by a trained dietitian. Also, physical activity (PA) for each individual was measured by face-to-face interviews using the International Physical Activity Questionnaire (IPAQ), which validated for the Iranian adult population [33]; finally, the results of this questionnaire were reported as Metabolic Equivalents per week (METs/week) [34, 35].

A socioeconomic status (SES) score [36], was calculated based on 5 dichotomous variables with score points of 0 and 1 including education (academic = 1, non-academic education = 0), family size (≤ 4 people = 1, >4 people = 0), income (high = 1, moderate and low = 0), house acquisition (house ownership = 1, without personal housing = 0), and foreign travel (yes = 1, no = 0). Then, the total SES score was computed by summing up the assigned scores (minimum SES score of 0 to maximum score of 5). Where an SES score of 3, 4, and 5 equated to high, 2 was scored as moderate, and 1 or 0 was scored as low.

### Statistical analysis

Data analysis was performed by Statistical Package Software for Social Science, version 21 (SPSS Inc., Chicago, IL, USA). The Kolmogorov-Smirnov test and histogram chart were used to assess the distributions of the variables. Information on the study population’s general characteristics and dietary intake were expressed for quantitative and qualitative variables as mean (standard deviation) or median and frequency (percent), respectively. To compare differences between the two groups (case and control groups), the independent-sample t-test and chi-square test was used for continuous and categorical variables, respectively. Individuals were categorized according to tertiles of dietary AGEs cut-points. Linear regression and Chi-square were used to test the trends of continuous and categorical variables across tertiles of dietary AGEs, respectively. Univariate analysis was performed for any confounding variable related to the NAFLD for selecting the potential confounders’ factors, then variables whose p-value was lower than 0.20 were considered confounders [37]. Also, the potential confounding variables in this study were selected based on the literature review and the variables that were considered as confounding in previous similar studies [23, 38]. In the multivariable model, the potential confounding variables include age, sex, BMI, smoking, PA, marital status, SES, and dietary intake of energy. A logistic regression test assessed the relationship between tertiles of dietary AGEs with odds of NAFLD. The odds ratios (ORs) with 95% confidence intervals (CIs) of NAFLD across tertiles of the AGEs were reported, and P-values < 0.05 were considered to represent statistical significance.

## Results

The mean age of all individuals (53% men) in this study was 38.13 ± 8.85 years. The mean BMI in participants was 26.85 ± 4.31 kg/m^2^. Also, the mean age in NAFLD patients (case group) and healthy subjects (control group) was 38.63 ± 8.71 and 37.88 ± 8.91 years, respectively. The median score of dietary AGEs intakes in individuals in the case and control groups was 4186 and 2798, respectively.

The participants’ characteristics across tertiles of dietary AGEs intake were reported in Table 1. Our results showed that the mean BMI of participants in the highest tertile of dietary AGEs was higher than those in the lowest tertile of AGEs, whereas the mean physical activity in subjects in the third tertile of dietary AGEs intake was lower than those in the first tertile of AGEs intake (P < 0.05). The percentage of individuals with high family size (more than four members), individuals with high income, and high SES was higher in the highest tertile of dietary AGEs intake compared to the lowest one (P < 0.05). However, there was no significant difference in other variables among participants across tertiles of dietary AGEs intake. Also, based on Table 1, the intakes of protein, total dietary fat, saturated fatty acid, monounsaturated fatty acid, and polyunsaturated fatty acid were increased across tertiles of dietary AGEs intake (P < 0.05), but the intakes of carbohydrate and fiber were reduced according to tertiles of dietary AGEs intake (P < 0.05).

**Table 1:** Study population characteristics across tertiles of dietary advanced glycation end products (per 1000 Kcal)

	T1 (n = 225)	T2 (n = 225)	T3 (n = 225)	P for trend*
*Demographic data*				
Age,(year)	38.7 ± 8.7	38.1 ± 8.9	37.6 ± 9.0	0.178
Male, (%)	49.8	52.4	56.9	0.312
Body mass index (Kg.m^2^)	25.2 ± 3.3	27.1 ± 4.6	28.1 ± 4.3	**< 0.001**
Physical activity ( MET/min/week)	1579 ± 976	1443 ± 867	1277 ± 764	**< 0.001**
Smoking (yes, %)	2.7	4.4	5.3	0.352
Marital status (married, %)	82.7	83.1	85.8	0.179
Education level (Bachelor and higher, %)	46.2	48.9	45.3	0.734
Family size (> 4 members, %,)	56.4	68.9	75.1	**< 0.001**
House acquisition (yes, %)	69.3	65.8	70.2	0.562
Foreign travel (yes, %)	13.8	13.8	20.0	0.113
Income (high, %)	6.7	9.3	18.2	**< 0.001**
Socio economic status (%)				**0.027**
Low (%)	30.7	28.4	25.3	
Middle (%)	43.1	37.8	33.8	
High (%)	26.2	33.8	40.9	
**Dietary intake**				
Energy intake(Kcal/d)	2244 ± 630	2181 ± 622	2229 ± 615	0.910
Carbohydrate (% of energy)	58.0 ± 7.2	56.9 ± 5.6	52.8 ± 6.6	**< 0.001**
protein(% of energy)	12.6 ± 1.9	13.5 ± 1.8	13.7 ± 2.9	**< 0.001**
fat(% of energy)	29.3 ± 7.3	29.7 ± 5.4	33.5 ± 6.9	**< 0.001**
Polyunsaturated fatty acids(% of energy)	6.3 ± 2.3	6.0 ± 1.8	7.0 ± 2.5	**0.001**
Monounsaturated fatty acids(% of energy)	10.2 ± 3.0	10.2 ± 2.1	11.6 ± 2.8	**< 0.001**
Saturated fatty acids(% of energy)	9.3 ± 3.0	10.3 ± 2.5	11.5 ± 3.0	**< 0.001**
Fiber (g/1000 Kcal)	17.5 ± 7.9	16.3 ± 6.4	14.6 ± 6.8	**< 0.001**

Data were expressed as mean ± SD and percent (%) for continuous and categorical variables, respectively

* P- Values were determined using linear regression and the chi-square test for continuous and categorical variables

The study population characteristics in case and control groups are shown in Table 2. Compared to subjects in the control group, participants in the case group had higher mean BMI and lower mean physical activity (P < 0.05). Also, the percentage of individuals with high family size (more than four members), high income, high SES, married individuals, or smokers was higher in the case group compared to the control group (P < 0.05). However, no significant difference was observed in the mean age, percentage of men, and education level of participants between the case and control groups. Table 2 reported that in comparison to the control group, the NAFLD patients group had a higher intake of energy (P = 0.004); however, no significant difference was found in macronutrient intakes between the case and control groups.

**Table 2:** Study population characteristics based on the case and control groups

	Non-NAFLD (n = 450)	NAFLD (n = 225)	P-value*
*Demographic data*			
Age,(year)	37.9 ± 8.9	38.6 ± 8.7	0.296
Male, (%)	51.8	55.6	0.354
Body mass index (Kg/m^2^)	25.0 ± 3.0	30.6 ± 4.0	**< 0.001**
Physical activity ( MET/min/week)	1590 ± 949	1119 ± 616	**< 0.001**
Smoking (yes, %)	2.7	7.1	**0.006**
Marital status (married, %)	81.3	88.9	**0.022**
Education level (Bachelor and higher, %)	47.8	44.9	0.478
Family size (> 4 members, %,)	57.3	85.8	**< 0.001**
House acquisition (yes, %)	71.8	61.8	**0.008**
Foreign travel (yes, %)	12.0	23.6	**< 0.001**
Income (high, %)	4.7	24.9	**< 0.001**
Socio economic status (%)			**< 0.001**
Low (%)	31.6	21.3	
Middle (%)	40.4	33.8	
High (%)	28.0	44.9	
**Dietary intake**			
Energy intake(Kcal/d)	2170 ± 625	2312 ± 606	**0.004**
Carbohydrate (% of energy)	55.9 ± 6.6	56.0 ± 7.4	0.913
protein(% of energy)	13.2 ± 2.2	13.2 ± 2.5	0.924
fat(% of energy)	30.8 ± 6.5	30.8 ± 7.4	0.939
Polyunsaturated fatty acids(% of energy)	6.3 ± 2.2	6.7 ± 2.5	0.193
Monounsaturated fatty acids(% of energy)	10.6 ± 2.6	10.8 ± 2.9	0.617
Saturated fatty acids(% of energy)	10.5 ± 2.9	10.2 ± 3.0	0.260
Fiber (g/1000 Kcal)	15.9 ± 6.5	16.8 ± 8.3	0.121

Data were expressed as mean ± SD and percent (%) for continuous variables and categorical variables, respectively

* P- Values were determined using the independent-samples t-test and the chi-square test for continuous and categorical variables, respectively

The ORs and 95% CIs for NAFLD across tertiles of AGEs are reported in Table 3. In the crude model, a significant association was observed between dietary AGEs intakes and risk of NAFLD (OR: 15.90; 95%CI: 9.30–27.30, P for trend < 0.001). Also, in model 2, after adjusting for age and sex, the odds of NAFLD were increased across tertiles of dietary AGEs intakes (OR: 16.48; 95%CI: 9.57–28.40, P for trend < 0.001). Also, in the final model, additionally adjusting for BMI, smoking, physical activity, marital status, SES, and dietary intake of energy, the odds of NAFLD in the highest tertile of dietary AGEs intakes were higher than those in the lowest one (OR: 12.16; 95%CI: 6.06–24.39, P for trend: <0.001). Furthermore, Table 3 showed that in the final model, after controlling the potential confounders, for each SD increase in dietary AGEs score, the risk of NAFLD increases more than 2.5 times (OR: 2.69; 95%CI: 2.02–3.58, P-Value < 0.001).

**Table 3:** Odds ratios (ORs) and 95% confidence intervals (CIs) for NAFLD based on tertiles of dietary advanced glycation end products (AGEs)

	OR(95% CI) of NAFLD					
	Tertiles of Dietary AGEs			P for trend	Per one SD	P-value
	T1	T2	T3			
**AGEs**						
Median score	2214	3262	4927	-	-	-
Case/Total	19 / 225	72 / 225	134 / 225	-	-	-
Crude model	1.00 (Ref)	5.10 (2.95–8.81)	15.90 (9.30–27.30)	< 0.001	2.83(2.28–3.50)	< 0.001
Model 1*	1.00 (Ref)	5.19 (2.99–8.98)	16.48(9.57–28.40)	< 0.001	2.85(2.30–3.53)	< 0.001
Model 2^†^	1.00 (Ref)	3.40 (1.68–6.88)	12.16 (6.06–24.39)	< 0.001	2.69(2.02–3.58)	< 0.001

^*^ Model 1: adjusted for age and sex

^†^ Model 2: adjusted for model 1 and body mass index, smoking, physical activity, marital status, socio-economic status, and dietary intake of energy

## Discussion

In this case-control study, we observed a remarkable positive relationship between high dietary AGEs intake and the risk of NAFLD independent of potential confounders, including age, sex, BMI, smoking, physical activity, marital status, SES, and dietary energy intake.

To the best of our knowledge, this is the first study have assessed the possible role of dietary AGEs intake in the odds of NAFLD in the adult population; however, our results are in line with the evidence from other epidemiological studies, which showed that this harmful food component could play an important role in predicting the increased risk of metabolic disorders, including cancer, hypertension, and MetS [23, 24, 39]. Mirmiran et al. study reported that a low intake of dietary AGEs in the framework of a healthy diet can reduce the risk of hypertension and its complications in Iranian adults [23]. Also, a study on Mexican adults suggests that a diet with a high intake of AGEs may be associated with an increased risk of hyperglycemia and MetS [24]. Furthermore, Wada et al., in a large observational study on women ≥ 35 years old, showed that a higher intake of dietary AGEs was positively related to the risk of liver cancer in Japanese men [39]. Therefore, both the previous evidence and our results confirm that a higher intake of AGEs in the diet has the potential to harm people’s health and increase the risk of chronic diseases, including liver diseases. However, due to the limitations of epidemiological studies, more studies are needed to investigate the severity of the relationship between this harmful food factor and the risk of NAFLD in other populations.

Although studies so far have not directly investigated the relationship between dietary AGEs intake and the risk of NAFLD, however, the results of our study can be explained and justified indirectly with the findings of studies that examined the relationship between serum AGEs, its receptors, or its products in metabolic pathways and liver disorders. Previously it showed a devastating effect of dietary or intra-peritoneal administered AGEs on liver function [40–42]. Hyogo et al. [43] reported that serum levels of AGEs increased in patients diagnosed with NASH compared to healthy controls. Also, it has been reported that steatosis caused by NAFLD was associated with serum AGEs levels in a positive manner [44]. Also, a case-control study demonstrated that AGEs and their receptor (RAGE) are culprits in liver damage [45]. Furthermore, according to another study by Priken [46] et al., elevated levels of hepatic AGEs are associated with increasing severity of NAFLD.

Binding AGEs to RAGE is the main mechanism by which, AGEs exert their effects that may play an important role in the initiation of liver damage or its acceleration by interfering with inflammatory and oxidative pathways. Several signal pathways are involved when the AGEs-RAGE axis is activated [47]. This, in turn leading to the production of reactive oxygen species (ROS) [48], which consequently triggers the activation of the Nuclear factor kappa B (NF-κB) pathway [49]. Thus, a vicious circle is formed since activation of NF-κB caused upregulation in RAGE production and maintained its effects. One of these effects includes consistent ROS production, which leads to AGEs formation and, therefore, gives rise to oxidative stress and more RAGE invigoration [50]. Leung et al. [42] have reported that the expression of RAGE was least possible in healthy people, but it is up-regulated in patients with fatty liver disease. When oxidative stress happens, the antioxidant system may be insufficient, and biomolecules are going under chemical changes due to ROS production, which leads to the inactivation of biomolecules and induces redox-sensitive transcription factors. As a result, pro-inflammatory and fibrogenic agents are produced by Kupffer and hepatic stellate cells [51]. Total oxidative stress is one of the main contributors to NAFLD risk [52].

Moreover, glycation of lipids, proteins, and nucleic acids resulted in AGEs formation [53, 54]. Generally, animal-based foods rich in fats and proteins have more AGEs content and tend to form a higher amount of new AGEs during the cooking process, in opposition, foods rich in carbohydrates have fewer AGEs [32]. This is in line with the present study’s findings that participants in higher tertiles of AGE intake consumed more proteins and fats. On the other hand, higher AGE intake is associated with lower carbohydrate consumption. There are some strategies by which, we can decrease the formation of new AGEs that including lower temperature, higher moisture, and making an acidic environment during the time of cooking [22].

The present study is the first to assess the association of dietary GEs with the risk of NAFLD in Iranian adults. Also, we used a validated and reproducible FFQ for individuals’ dietary intake assessment. Furthermore, data on physical activity was determined using a validated questionnaire. In addition, there are some limitations; first, the case-control nature of this study disabled us to clarify the cause-and-effect relationship. Second, using a questionnaire to collect food and physical activity data makes measurement errors inevitable; however, we used a validated questionnaire for our population to reduce possible errors. Third, we did not collect data on the different cooking processes, which can affect the AGEs content of foods. Furthermore, the present hospital-based case-control study was performed in a single metabolic liver disease center in Iran (affiliated with Isfahan University of Medical Sciences), which may limit its generalizability; however, it should be noted that this center was a referral clinical center, whose clients could be from all different parts of Isfahan city.

## Conclusions

Our findings revealed that higher adherence to a dietary pattern with high AGEs score is associated with increased odds of NAFLD. This is an interesting result since it can help define a dietary pattern, which focuses on dietary components that have no or very little AGEs and can be easily adhered to by the public to prevent growing poor health outcomes such as liver disorders. Also, it is recommended to perform epidemiological studies, especially prospective studies with a higher sample size and long-term follow-up to confirm the evidence regarding the possible relationship between dietary AGEs intake and the risk of NAFLD.

## Data Availability

The datasets analyzed in the current study are available from the corresponding author on reasonable request.

## Abbreviations

AGEs: Dietary advanced glycation end products
BMI: body mass index
CIs: confidence intervals
FCT: Food Composition Table
FFQ: food frequency questionnaire
IPAQ: International Physical Activity Questionnaire
METs: Metabolic Equivalents
MetS: metabolic syndrome
NAFLD: Nonalcoholic fatty liver disease
NF-Κβ: Nuclear factor kappaβ
ORs: odds ratios
PA: physical activity
RAGE: Receptor for AGE
SES: socioeconomic status
SPSS: Statistical Package Software for Social Science
USDA: United States Department of Agriculture
